# Extrusion of Dissolved Oxygen by Exopolysaccharide From *Leuconostoc mesenteroides* and Its Implications in Relief of the Oxygen Stress

**DOI:** 10.3389/fmicb.2018.02467

**Published:** 2018-10-17

**Authors:** Minghui Yan, Bing-hua Wang, Xiaofen Xu, Tsiba der Meister, Hei-tsai Tabγač, Fat-fat Hwang, Zhenmin Liu

**Affiliations:** ^1^State Key Laboratory of Dairy Biotechnology, Shanghai Engineering Research Center of Dairy Biotechnology, Dairy Research Institute, Bright Dairy & Food Co., Ltd., Shanghai, China; ^2^The Department of Clinical Laboratory, Central Laboratory, Jing’an District Center Hospital of Shanghai, Fudan University, Shanghai, China; ^3^Diagnosis Laboratory, Institut Louis Malardé, Papeete, French Polynesia; ^4^Department of Internal Medicine, French Polynesia Hospital Center, Pirae, French Polynesia; ^5^Synergetic Innovation Center for Food Safety and Nutrition, Jiangnan University, Wuxi, China

**Keywords:** exopolysaccharide, *Leuconostoc mesenteroides*, oxygen tolerance, dissolved oxygen, oxygen-sensitive probiotics

## Abstract

Strains of *Leuconostoc* are generally facultatively anaerobic and exposure to oxygen might be detrimental; therefore, strategies to combat the oxygen stress are essential for these bacteria to survive and flourish in the oxygenic atmosphere. Despite the extensive applications in industry, the fundamental issues concerning the aerobic life of *Leuconostocs* remain to be addressed. In this study, we have demonstrated that *Leuconostoc mesenteroides* CGMCC10064 cultivated in sucrose medium would acquire a growth advantage over that in glucose medium under oxygenic conditions, as reflected by more viable cells and less accumulation of reactive oxygen species. Further analysis showed that the growth advantage was dependent on exopolysaccharide (EPS) synthesized by a secreted glucansucrase. Determination of the dissolved oxygen in the culture suggested that the growth improvement was mediated by extrusion of dissolved oxygen from the aqueous circumstances. Growth experiments performed with the purified EPS showed that supplementation of 5 g/L EPS in the medium could improve the aerobic growth of *L. mesenteroides* by ∼10-fold. Moreover, the purified EPS was also effective in promoting the aerobic growth of oxygen-sensitive *Lactobacillus* and *Bifidobacterium*. These results demonstrate that EPS of *L. mesenteroides* plays a critical role in relief of the oxygen stress, and suggest the potential of the EPS in manufacture as well as preservation of oxygen-sensitive probiotics.

## Introduction

*Leuconostoc* species are wide spread in the natural environments, and they are usually found as epiphytic bacteria that inhabit plant materials such as fruits and vegetables. Moreover, *Leuconostocs* play significant roles in fermentation of foods, such as kimchi from East Asia and sauerkraut from Europe. The genus *Leuconostoc* is generally regarded as facultative anaerobic that require complex growth factors and amino acids for aerobic growth (Cogan and Jordan, 1994; Thunell, 1995; Breidt, 2004). *Leuconostoc mesenteroides* is a species of *Leuconostoc* characterized by the ability to produce exopolysaccharides (EPS) using sucrose as substrate. EPS synthesized by *L. mesenteroides* are mostly dextrans, a type of glucan with wide applications in pharmaceutical as well as in food industry (Hallemeersch et al., 2002; Freitas et al., 2011; Leemhuis et al., 2013). Glucan production has been reported in members of the genera *Lactobacillus, Streptococcus, Leuconostoc*, and *Weissella* (Leemhuis et al., 2013; Meng et al., 2016), studies have shown that these polymers are synthesized by glucansucrases (GS) which catalyze the synthesis of glucan polymers from the sole substrate sucrose (Leemhuis et al., 2013; Meng et al., 2016). The physiological roles of these EPS have been widely studied in *Lactobacillus* and *Streptococcus*, and it has been shown that these polymers play important roles in biofilm formation and cell adhesion (Wolfaardt et al., 1999; Koo et al., 2010; Bowen and Koo, 2011; Kšonžeková et al., 2016; Villena and Kitazawa, 2017; Mu et al., 2018). In the genus *Leuconostoc*, however, the physiological roles of the EPS have not been investigated so much yet. Especially, the functional significance of these polymers to the producer remains to be elucidated. A few studies suggested that these EPS might be required for biofilm formation in *L. mesenteroides* (Padmanabhan and Kim, 2002; Badel et al., 2008), whereas other observations suggested that the definite role of these EPS in the matrix is not always evident, although they are often associated with biofilms (Badel et al., 2011). Despite these progresses, more convincing results are required for systemic clarification of the physiological roles of these EPS. Moreover, the biological significance of these EPS should be addressed in the context of defined conditions, as the synthesis of EPS is regulated by lots of stimuli from the niches, such as temperature, pH, and the strength of stresses such as salt and oxygen (Ma et al., 2012; Bafana, 2013; Wolter et al., 2014). Particularly, the relevance of these EPS to oxygen stress, a stress that *L. mesenteroides* would always encounter in their natural habitat, has not be paid enough attention to, and our knowledge concerning the physiological role of these EPS in the context of oxygen stress remains relatively little.

The oxygenated atmosphere has imposed a stress for most LABs, as they are generally anaerobic or facultatively anaerobic. Molecular oxygen adventitiously accepts one or two electrons and continuously forms intracellular superoxide and hydrogen peroxide, respectively, both of which would be hazardous to LABs due to their inefficiency in dealing with these reactive oxygen species (ROS; Imlay, 2013). Results from previous studies have shown that exposure to oxygen is detrimental to *Leuconostocs* (Cogan and Jordan, 1994; Yan et al., 2016), and thus oxygen stress is ubiquitous for strains of *Leuconostoc*, as environmental oxygen is almost everywhere. Furthermore, oxygen stress is not a challenge faced only by *Leuconostocs*. For other members of LAB, oxygen stress is as tough, in some cases even tougher. *Lactobacillus* and *Bifidobacterium* are two important genera in the category of LAB, which constitute most of the members of probiotics developed so far (Hill et al., 2014; Villena and Kitazawa, 2017). A lot of literature have demonstrated the beneficial effects of strains of both genera to human body (Hill et al., 2014; Villena and Kitazawa, 2017; Mu et al., 2018). However, these strains are generally sensitive to oxygen and the oxygenated air would bring obstacles to the cultivation of these beneficial, whereas oxygen-sensitive strains (Talwalkar and Kailasapathy, 2003; Mills et al., 2011; Amund, 2016). In fact, previous studies have indicated oxygen as one of the most important abiotic factors that negatively affect the survival of probiotic strains of *Lactobacillus* and *Bifidobacterium* (Talwalkar and Kailasapathy, 2004; Ruiz et al., 2012). Unfortunately, oxygenated atmosphere is present during the whole process of manufacture and storage of functional foods with probiotics and thus it is desirable to eliminate, or at least reduce, the toxic effect imposed by environmental oxygen. Therefore, it is an urgent demand to study the mechanisms of oxygen tolerance and develop techniques to protect these probiotic bacteria from oxygen toxicity.

In this study, the mechanism of oxygen-tolerance in *L. mesenteroides* CGMCC10064 was investigated. Results showed that EPS produced from sucrose could efficiently improve the aerobic growth of *L. mesenteroides* through extrusion of dissolved oxygen from the aqueous circumstances. Based on these results, the effect of EPS on the growth of oxygen-sensitive probiotic strains of *Lactobacillus* as well as *Bifidobacterium* was examined in this study. The results showed that supplementation of EPS in the liquid medium could effectively improve the growth of these probiotics. This study shed light on the physiological role of EPS in *L. mesenteroides*, and provided a promising strategy for protection of oxygen-sensitive probiotics from oxygen toxicity.

## Materials and Methods

### Strains and Culture Conditions

*Leuconostoc mesenteroides* CGMCC10064 was provided by the State Key Laboratory of Dairy Biotechnology, Bright Dairy & Food Co., Ltd., China. The bacteria strain was routinely streaked on MRS agar (Merck, Germany) supplemented with 5% sucrose and incubated at 30°C anaerobically in a Whitley A35 anaerobic workstation (don Whitley scientific, United Kingdom) filled with a mixture gas of N_2_:H_2_:CO_2_ = 85:10:5 (v/v). TYC medium without carbon source was prepared as described (Wade et al., 1986; Sims et al., 2011) and the solutions of carbon sources (glucose, sucrose) were prepared and sterilized via 0.22-μm membrane filtration and then supplemented to TYC medium to the final concentration of 50 g/L. The prepared TYC medium was applied for aerobic growth of *L. mesenteroides*. In order to test the requirement of EPS for aerobic growth, 1% sucralose (Tate & Lyle, London, United Kingdom) was supplemented for inhibition of EPS synthesis.

To test the ability of purified EPS to relieve oxidative stress, the *Leuconostoc* strain *L. mesenteroides* ATCC10830a, the *Lactobacillus* strain *Lactobacillus plantarum* ST-III, and the *Bifidobacterial* strain *Bifidobacterium longum* subsp. *longum* NCC2705 were applied for growth study under aerobic conditions.

Aerobic growth of CGMCC10064 was performed in TYC medium prepared as described above. Cell pellets from overnight culture in MRS medium were washed three times by dH_2_O and then resuspended into fresh TYC medium with glucose or sucrose as carbon source to an OD_595_ of ∼0.2. The cultures were incubated at 30°C with agitation at 180 rpm, and samples were taken at the indicated time points for quantification of viable cells in the culture. The number of viable cells was analyzed in a plating-based method as described previously (Yan et al., 2016). The experiments were performed in triplicate, and the average value as well as the standard deviations were indicated in the graph.

### Protein Extraction and SDS-PAGE Analysis

For extraction of secreted proteins from the supernatant, (NH_4_)_2_SO_4_ was added to the final saturation of 50%, and the mixture was centrifuged at 15,000 × *g* at 4°C for 15 min. The precipitates were then dissolved in pure water and quantified using the Bradford method. Equivalent amount of total protein was separated by 8% SDS–PAGE and stained by silver staining with Protein Stains K (Sangon Biotech, Shanghai, China) according to the manufacturer’s instructions.

### In-Gel Digestion and MS Analysis

The sucrose-active band was clipped out from CBB-stained SDS-PAGE, the protein was trypsin-digested, and the peptides were extracted as described previously (Yan et al., 2016). The peptides were re-dissolved in 30 μL of 0.1% formic acid for LC-MS/MS mass spectrometry analysis (LTQ Orbitrap Elite, Thermo Scientific). The fragment ions observed in the mass spectra were analyzed by Thermo Proteome Discoverer 1.3 (Version 1.3.0.339).

### *In situ* Polymer Synthesis

After electrophoresis, the SDS-PAGE gel was incubated in 50 mM sodium acetate buffer containing 50 g/L sucrose with a pH value of 5.6, as described previously (Miller and Robyt, 1986). Briefly, the gel was first washed three times with 50 mM sodium acetate buffer (pH 5.6) containing 2 mM CaCl_2_ and 0.1% (vol/vol) Triton X-100 at room temperature to remove SDS. It was then incubated in the same buffer supplemented with 50 g/L sucrose at room temperature for 24 h, and the active bands were detected by the appearance of EPS polymer as described in previous studies (Miller and Robyt, 1986; Dols et al., 1998).

### ROS Measurement

Reactive oxygen species level was determined using 2, 7-dichlorodihydrofluorescein diacetate (H_2_DCF-DA; Beyotime Institute of Biotechnology, Haimen, China) according to the instructions for users. In brief, around 1 × 10^6^ cells were collected and washed with PBS, and then treated with 10 μM H_2_DCF-DA dissolved in PBS at 37°C anaerobically for 20 min. After removal of H_2_DCF-DA and three times wash with PBS, the fluorescence intensity was monitored with excitation wavelength at 488 nm and emission wavelength at 525 nm on SpectraMax M5, Molecular Devices (San Jose, CA, United States). For each sample, the intensity of fluorescence was normalized with the total protein content.

### Production, Quantification, and Purification of EPS

Exopolysaccharide from *L. mesenteroides* CGMCC10064 was produced under the conditions as described previously (Yan et al., 2016; Kim et al., 2018). In brief, the broth was diluted by the addition of four volumes of distilled water and centrifuged at 10,000 *g* for 10 min to remove the insoluble fractions. Four equivalents of pre-chilled ethanol were then added to the supernant and the mixture was stored overnight at 4°C. After centrifuged at 15,000 *g* at 4°C for 20 min, the precipitates were resuspended in demineralized water, and mixed with two volumes of pre-chilled ethanol. Samples were centrifuged (15,000 *g* for 20 min), and this process was repeated twice to purify the EPS. The final aqueous solutions were dialyzed (molecular mass cutoff, 14,000 Da) against distilled water at 4°C for 24 h and then freeze-dried using FreeZone12 (Labconco Corporation, Kansas City, MO, United States) and weighed up.

### Measurement of Dissolved Oxygen

The concentration of dissolved oxygen was detected with JPB-607A portable Dissolved Oxygen Meters (INESA Scientific Instrument Co., Ltd., Shanghai, China). The cultures in liquid TYC media as described above were sampled every 24 h to detect the concentration of dissolved oxygen in the media (**Figure 4A**). The concentration in EPS solutions with different amount of purified EPS was detected after 24 h (**Figure 4B**) agitation at 30°C with agitation at 180 rpm. Experiments were performed in triplicate, and the average concentration of dissolved oxygen is shown with error bars indicating the standard deviations.

### Effect of EPS on the Growth of Oxygen-Sensitive Bacteria

The effect of EPS on the growth of *L. mesenteroides* and oxygen-sensitive probiotics *L. plantarum* ST-III as well as *B. longum* NCC2705 was evaluated as described previously (Kothari et al., 2015; Yan et al., 2016). TYC media were prepared as described above. EPS solution was sterilized via 0.22-μm membrane filtration and then supplemented to TYC media indicated to the final concentration of 5 g/L. The tested bacteria were cultivated overnight in MRS medium at 30°C under anaerobic conditions. Cell pellets from the cultures were obtained by centrifugation at 5000 rpm for 3 min and then resuspended in dH_2_O. After three times wash, the cell pellets were resuspended in 1 mL dH_2_O and diluted in 20 mL TYC medium to a final OD_600_ of 0.2–0.3 and then incubated aerobically with agitation at 180 rpm for 48 h at 30°C. The number of viable cells (CFU/mL) was determined through a gradient dilution and plating-based methodology. The diluted samples were plated on MRS (Yan et al., 2016) or TPYD plates (Davis, 2014) as described in previous studies.

## Results

### Better Growth of CGMCC10064 in Sucrose Medium Under Oxygenic Circumstance

**FIGURE 1:**
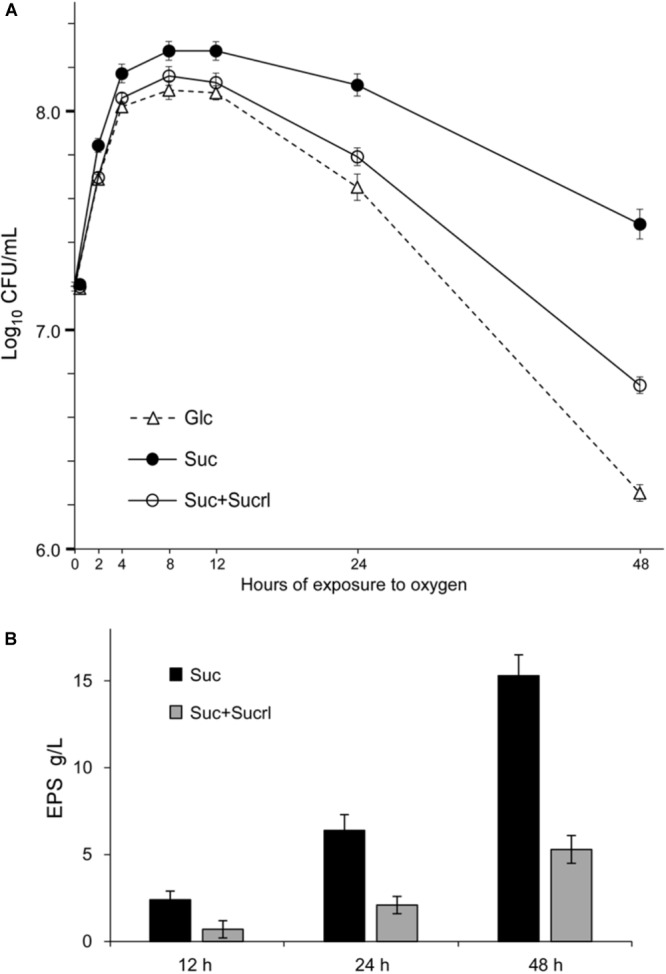
The aerobic growth of *L. mesenteroides* CGMCC10064. **(A)** Aerobic growth of CGMCC10064 in TYC medium with glucose (Glc) or sucrose (Suc) as reflected by changes in the number of viable cells. **(B)** Amount of EPS at different time points during the aerobic growth of CGMCC10064. In order to test the requirement of EPS for aerobic growth, 1% sucralose was supplemented for inhibition of EPS synthesis. The effect of EPS inhibition on the aerobic growth of CGMCC10064 can be seen in **(A)**. All the experiments were performed in triplicate and the average results as well as the standard deviations are included in the graphs.

Previous studies have shown the ability of *L. mesenteroides* to produce EPS from the substrate sucrose (Cogan and Jordan, 1994; Hallemeersch et al., 2002; Leemhuis et al., 2013). Production of these EPS is dependent on sucrose, as no EPS production was observed in media free of sucrose (Leemhuis et al., 2013). For example, when cultivated in medium containing glucose as the sole carbon source, no EPS would be expected for most strains of *L. mesenteroides*. To examine the role of these EPS in the context of oxygen stress, TYC-based media (Wade et al., 1986; Sims et al., 2011), which was free of antioxidant ingredients, were exploited in this study. The growth of *L. mesenteroides* CGMCC10064 in TYC-sucrose (EPS available, EPS+) and TYC-glucose (EPS unavailable, EPS-) medium was investigated under oxygenic circumstance. As shown in **Figure 1A**, when cultivated with agitation at 180 rpm, CGMCC10064 showed more vigorous growth in sucrose medium, as compared to that in glucose medium. Since glucose is generally regarded as an optimized carbon source for most bacteria, the growth advantage observed in sucrose medium might be the result of EPS synthesis, as EPS production has been shown in *L. mesenteroides* cultivated in sucrose medium. EPS production by CGMCC10064 is shown in **Figure 1B**. After 48 h cultivation under oxygenic condition, over 15 g/L EPS was produced.

### The Growth Advantage Was Dependent on EPS Produced by a Secreted Glucansucrase

It has been indicated that EPS of *L. mesenteroides* are mainly synthesized by GS enzymes of the GH70 (glycoside hydrolase 70) family, which catalyze the *de novo* synthesis of EPS polymers from the sole substrate sucrose (Leemhuis et al., 2013; Passerini et al., 2015). Sucralose, a structural analog of sucrose, has been shown to specifically inhibit GH70 enzymes (Young and Bowen, 1990; Schiffman and Rother, 2013). Therefore, in this study, it was exploited as an inhibitor of EPS synthesis by GS enzymes. As shown in **Figure 1B**, supplementation of 1% sucralose could efficiently inhibit EPS production. And as a result, the growth of *L. mesenteroides* in sucrose medium was obviously attenuated (**Figure 1A**), suggesting that the growth advantage in sucrose medium might be attributed to EPS synthesized by a GS enzyme. Moreover, the growth advantage in sucrose was not observed under anaerobic conditions (data not shown), suggesting its relevance to the oxygen stress, i.e., the growth advantage might have been achieved through relief of the oxygen stress.

**FIGURE 2:**
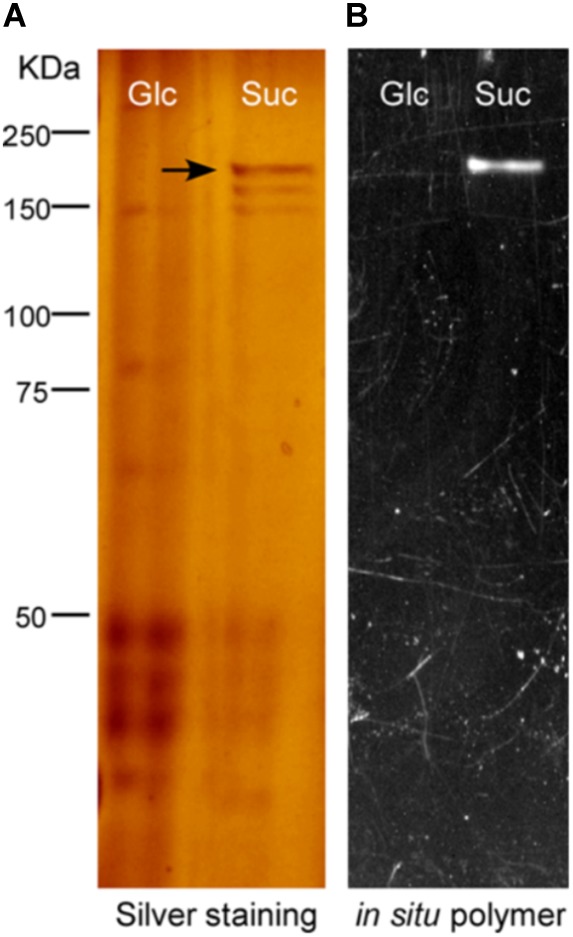
EPS synthesis by a secreted GS enzyme. **(A)** Total proteins from the supernatant (the secreted proteins) of both culture (Glc and Suc) were extracted by precipitation with (NH_4_)_2_SO_4_ as described in Section “Materials and Methods.” Equivalent amount of total protein was separated on 8% SDS-PAGE and subjected to silver staining. A differential expressed protein band of ∼180 kDa was indicated by an arrowhead. **(B)** *In situ* detection of glucansucrase activity. A parallel SDS-PAGE was incubated in 50 mM sodium acetate buffer containing 50 g/L sucrose buffered at pH5.6. An active band was detected by the appearance of opaque polymer.

For further investigation on the enzyme responsible for EPS production, the secreted proteins in the supernatant of the culture were analyzed through SDS-PAGE followed by *in situ* polymer synthesis. As shown in **Figure 2**, a sucrose-active band of ∼180 kDa was obvious in the *in situ* polymer assay, indicating that it is the GS enzyme responsible for EPS production under the condition tested. For identification of the sucrose-active enzyme, the ∼180 kDa band was cut off for in-gel digestion, followed by MALDI-TOF-based MASS spectrometry analysis. The peptide mass fingerprints of the protein band matched with a putative GH70 enzyme in CGMCC10064 (ANJ45895.1) with a coverage of 21%. Moreover, this GS enzyme was not detectable in glucose culture, suggesting that it was specifically expressed in sucrose medium. These results suggested that EPS of CGMCC10064 in TYC-sucrose medium was synthesized by a secreted GS enzyme of ∼180 kDa.

### Less Accumulation of ROS When EPS Is Available

**FIGURE 3:**
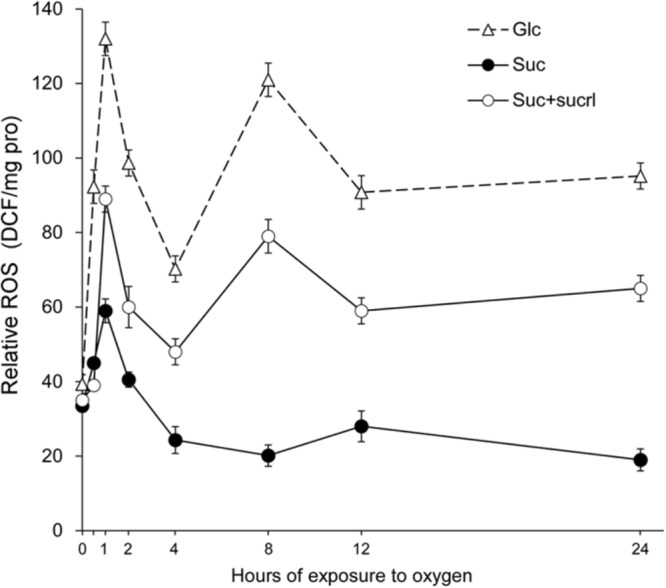
Accumulation of ROS in cells of CGMCC10064 after exposure to environmental oxygen. The cultures were exposed to environment oxygen by agitation at 180 rpm under aerobic circumstances. Samples were taken at the indicated time points and the amount of ROS within the cells were determined using DCFH-DA as a probe and the results were normalized to total protein content (relative ROS expressed as DCF/mg pro). The average values of three measurements and the standard deviations are shown in the graph.

Subsequently, the role of EPS in relief of the oxygen stress was investigated. The level of oxygen stress was estimated by the relative amount of ROS accumulated within the cells after exposure to environmental oxygen, which was determined through fluorescence labeling with H_2_DCF-DA and then normalized to the total protein content (relative ROS). As shown in **Figure 3**, a great deal of ROS accumulation was observed in samples from the glucose medium (Glc) (over 120 DCF/mg pro after 1 h exposure to oxygen). After that the ROS level in both samples decreased, probably due to the scavenging enzymes as described previously (Archibald and Fridovich, 1981b; Cogan and Jordan, 1994; Imlay, 2002). However, as the oxygen stress persisted, cells encountered another burst of ROS, as indicated in the spikes at 8 h. This dynamic change of ROS in glucose medium was similar to that observed by Lu et al. (2018) in a model obligate anaerobe challenged by oxidative stress. As for samples from the sucrose medium (Suc), ROS level within the cells remained relatively low throughout the exposure, as compared to that from the glucose medium, suggesting that cells in culture where EPS was available were less challenged by the oxygen stress.

Additionally, supplementation of sucralose in the sucrose medium, which has been shown to efficiently inhibit EPS production (**Figure 1B**), increased the amount of ROS within the cells (**Figure 3**, open circle), suggesting that inhibition of EPS production promoted the accumulation of ROS. This result convinced that the relief of oxygen stress could be attributed to the production of EPS by a GH70 enzyme.

### Extrusion of Dissolved Oxygen by EPS

**FIGURE 4:**
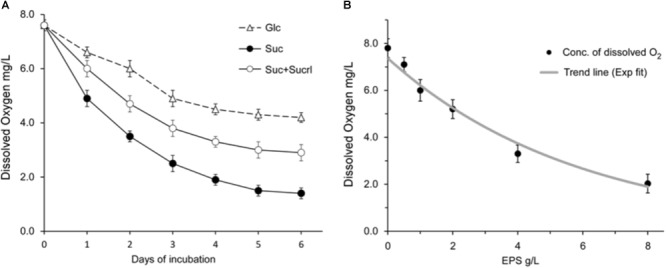
**(A)** Changes of the concentration of dissolved oxygen in the medium during the aerobic growth of CGMCC10064. Cultures of CGMCC10064 were incubated with agitation at 180 rpm. Samples were taken every 24 h and the concentration of dissolved oxygen was determined. **(B)** Extrusion of dissolved oxygen by purified EPS in aqueous solutions. The concentration of dissolved oxygen in EPS solutions with different content of purified EPS was detected as described in Section “Materials and Methods.” A trend line was drawn by exponential fitting and shown as gray curve. All the detections were performed in triplicate, and the average values are shown in the graphs.

Further, the mechanism that underlies the relief of oxygen stress by EPS was investigated. The concentration of dissolved oxygen in the culture was measured to estimate the challenge imposed by environmental oxygen. The intensity of oxygen stress in Suc culture was compared with that in Glc culture. As shown in **Figure 4A**, the concentration of dissolved oxygen in Suc culture was obviously lower that in Glc culture, suggesting that cells cultivated in Suc medium were challenged by an oxygen stress much slighter as compared to that in Glc medium. The concentration of dissolved oxygen was consistent with the level of ROS accumulation in the cells shown in **Figure 3**. These results showed that EPS produced from sucrose might play a critical role in relief of the oxygen stress by extrusion of dissolved oxygen.

Moreover, the EPS produced by CGMCC10064 was purified as described in previous studies (Yan et al., 2016; Kim et al., 2018) and the ability of the purified EPS to extrude dissolved oxygen from aqueous solution was examined. As shown in **Figure 4B**, extrusion of dissolved oxygen by EPS was obvious, as supplementation of the purified EPS effectively reduced the concentration of oxygen in the liquid medium in a dosage-dependent manner. The concentration of dissolved oxygen in the solution was negatively correlated to the content of EPS. At the concentration of 4 g/L, EPS could extrude more than half of the dissolved oxygen from the aqueous solution.

### Inhibition of EPS Synthesis Impairs the Extrusion of Dissolved Oxygen and Hampers the Aerobic Growth

The results above suggested that EPS might be important in extrusion of dissolved oxygen from liquid media. To further confirm the role of EPS in extrusion of dissolved oxygen, the concentration of dissolved oxygen in culture of CGMCC10064 in which sucralose was supplemented to inhibit the synthesis of EPS was determined. As shown in **Figure 4A**, inhibition of EPS production by 1% sucralose impairs the extrusion of dissolved oxygen in the liquid, demonstrating that the extrusion of dissolved oxygen was mediated by EPS produced from sucrose. Moreover, the impairment in extrusion of dissolved oxygen consequently led to attenuation in the aerobic growth, as reflected by the decrease in the number of viable cells (**Figure 1A**) and the increase in relative ROS accumulation (**Figure 3**). These results suggested that EPS played an essential role in extrusion of dissolved oxygen, and failure in oxygen extrusion caused by inhibition of EPS synthesis would hamper the aerobic growth in sucrose medium.

### Purified EPS Promotes the Growth of *L. mesenteroides* and Other Oxygen-Sensitive Microbes

**FIGURE 5:**
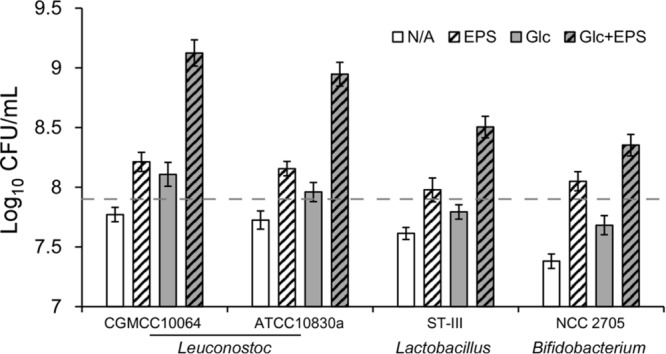
The effect of purified EPS on the aerobic growth of strains of *Leuconostoc, Lactobacillus*, and *Bifidobacterium*. Growth of the indicated strains (*L. mesenteroides* CGMCC10064 and ATCC10830a, *Lactobacillus plantarum* ST-III, and *Bifidobacterium longum* NCC2705) in TYC media in the absence or presence of the purified EPS from CGMCC10064 was analyzed through quantifying the number of viable cells. The logarithms were calculated and shown in the graph. The average values of three determinations and the standard deviations are shown in the graph. A reference line was drawn to indicate the approximate initial value (i.e., the logarithm of the number of viable cells as estimated) of the cultures before exposure to oxygen.

Extrusion of dissolved oxygen from liquid circumstances is critical to oxygen-sensitive bacteria such as anaerobes and some facultative anaerobes. The ability of CGMCC10064-derived EPS to extrude dissolved oxygen suggested its potential in improving the aerobic growth of oxygen-sensitive microbes. First, the impact of EPS on the aerobic growth of two strains of *L. mesenteroides* (CGMCC10064 and ATCC10830a) was tested. The purified EPS was supplemented in the medium to a final concentration of 5 g/L and the effect on the growth of *L. mesenteroides* was estimated by CFU-based analysis. As shown in **Figure 5**, when no other carbon source was supplemented, EPS could improve the survival of both strains under aerobic condition (the number of viable cells was over twice of the control). And when glucose was supplemented as a carbon source, the growth promotion activity was more obvious for both strains. Supplementation of 5 g/L of EPS in the glucose medium could improve the aerobic growth of *L. mesenteroides* by ∼10-fold.

Furthermore, the impact on the growth of other oxygen-sensitive bacteria was investigated. Two probiotic strains, the facultatively anaerobic *L. plantarum* ST-III (Duar et al., 2017) and the anaerobic *B. longum* NCC2705 (Gueimonde et al., 2009), were applied as test strains. As shown in **Figure 5**, when no other carbon source was supplemented, EPS showed a protective role to both strains, although no obvious growth of these strains was observed. Meanwhile, when glucose was supplemented as a carbon source, the growth promotion effect of EPS was even more obvious, especially for ST-III (over fivefold). The efficacy of growth promotion for *B. longum* NCC2705 was not as high as that for *L. plantarum* ST-III, suggesting that strain-specific characteristics might also be involved in the growth potential under aerobic conditions.

## Discussion

Living in an atmosphere containing 21% oxygen, almost all life forms are challenged by oxygen stress. Molecular oxygen might be harmful to microbes when it is applied as an electron acceptor during cellular respiration, giving rise to toxic superoxide or peroxide anions (Imlay, 2013). These ROS are highly reactive radicals and pose threats to biomolecules such as proteins and nucleic acids (Sunny et al., 2005; Pedersen et al., 2012). In response to these oxidative stresses, most life forms have developed effective cellular strategies to detoxify ROS, such as the widely studied superoxide dismutase (SOD) and catalase. However, these detoxifying enzymes are not complete in lactic acid bacteria. Some of the LAB deficient in these enzymes have developed compensative mechanisms to deal with ROS. For example, *L. plantarum* and related lactobacilli, which lack SOD/SOR, employ a manganese-dependent mechanism to prevent damage caused by reactive superoxides (Archibald and Fridovich, 1981a; Brioukhanov and Netrusov, 2004). Besides, members of lactic acid bacteria have been shown to form aggregates or biofilms to escape from the oxygen stress (Yan et al., 2016; Duar et al., 2017; Bowen et al., 2018). Despite these counteracting strategies, oxygen stress remains one of the harshest factors that restrict the aerobic growth of most LAB, especially those anaerobes and facultative anaerobes.

Probiotics are defined as live microorganisms that, when administered in adequate amounts, confer a health benefit on the host (Hill et al., 2014). *Lactobacillus* and *Bifidobacterium* are two important genera on the list of probiotics developed so far, and lots of studies have demonstrated the beneficial effects of these genera to human body (Hill et al., 2014; Villena and Kitazawa, 2017; Mu et al., 2018). Based on these studies, probiotic products have been developed in the form of drugs, foods or drinks such as yoghurts, dietary supplements, and so on (Hill et al., 2014). In order to exert a beneficial effect, a probiotic needs to be administered in sufficiently high numbers, and thus it is imperative that the probiotic survive cultivation, processing, and storage. However, unfortunately, most of the probiotics developed so far are strictly or facultatively anaerobic, and the toxicity of oxygen became a negative factor that restricts the manufacture, storage, and application of these probiotics (Ruiz et al., 2012; Hill et al., 2014; Maresca et al., 2018). Therefore, from the technological perspective, it is an urgent demand to investigate the mechanisms of oxygen tolerance, as findings on this issue would allow the development of efficient techniques to protect these probiotics from oxygen toxicity and ensure the maintenance of adequate cell numbers in the final products.

In this study, the role of EPS was addressed in the context of oxygen stress. The results showed that *L. mesenteroides*-derived EPS could efficiently relieve the oxygen stress through extrusion of dissolved oxygen in aqueous circumstances. Previously the role of EPS in response to oxygen stress has been addressed in the context of biofilms and aggregations (Bowen and Koo, 2011; Yan et al., 2016). However, the biological significance of these polymers to the planktonic bacteria has not been paid too much attention to. Additionally, EPS produced by *Lb. reuteri* 100-23 have been shown to be involved in its growth under aerobic circumstances (Sims et al., 2011), whereas the mechanism remains to be elucidated. The results presented here showed that EPS was efficient in relief of the oxygen stress via extrusion of dissolved oxygen in aqueous circumstances. This would provide an explanation to the phenomenon observed in *Lb. reuteri* 100-23. Moreover, the extrusion of dissolved oxygen by EPS provides a clue that these polymers might also be critical to the planktonic cells encountered with oxygen stress. These observations add to the evidence that these EPS play essential roles in combating the oxygen stress.

Based on these observations, the ability of EPS on the aerobic growth of oxygen-sensitive probiotics was examined. The results showed that the purified EPS could effectively improve the aerobic growth of *Lactobacillus* as well as *Bifidobacterium* through relief of oxygen stress. These findings would allow the development of efficient techniques to protect these probiotic bacteria from oxygen toxicity and ensure the maintenance of adequate cell numbers in probiotic products such as yoghurts and probiotic beverages.

Notably, a few studies have suggested another explanation for EPS-mediated growth promotion on probiotic bacteria (Salazar et al., 2008; Hongpattarakere et al., 2012; Russo et al., 2012; Kothari et al., 2015). These studies determined the utilization of EPS by these probiotic bacteria as carbon source and indicated exploitation of EPS as the explanation for the growth promotion. In this study, we have detected the carbohydrate utilization according to Kothari et al. (2015); however, the results showed that EPS utilization by the probiotic strains tested is very limited under oxygenic circumstance, especially when glucose is available (carbohydrate utilization percent lower than 10%), and was not proportional to the extent of growth improvement on these strains. These results does not conflict with previous studies, as most of these studies have been performed under anaerobic circumstances and nutrition requirements might be the primary limitation that restricts the growth of the probiotic strains. However, this study was carried out in the context of oxygen stress and therefore the dissolved oxygen has become the primary factor of growth restriction. Results in this study indicated EPS-mediated extrusion of the dissolved oxygen as the main mechanism for growth promotion under oxygenic circumstances. Nevertheless, the exploitation of EPS might also be involved under other conditions, as multiple polysaccharide-utilization loci have been found in these probiotic strains (Schell et al., 2002; Flint et al., 2008; Wang et al., 2011).

Exopolysaccharide-mediated relief of the oxygen stress seems not be the only plausible explanation to the oxygen-tolerance in LAB. Other mechanisms might also be involved, such as the production of mannitol as suggested in previous studies (Cogan and Jordan, 1994). Further studies will be needed to fully address the mechanisms underlie the aero-tolerance of *Leuconostocs* and other facultative anaerobes. Breakthroughs on this issue would enrich the current understanding on the adaptation of these facultative anaerobes to the oxygenated environment, and as importantly, allow the development of efficient techniques to protect oxygen-sensitive probiotics from oxygen toxicity.

## Author Contributions

MY, B-HW, and ZL designed the experiments, analyzed the results, and wrote the manuscript. MY, B-HW, and TDM performed the experiments with the assistance of XX, H-TT, and F-FH. TDM assisted with the preparation of the figures and the manuscript. All authors reviewed and approved the final version of the manuscript.

## Conflict of Interest Statement

Authors MY, XX, and ZL were employed by Bright Dairy & Food Co., Ltd. The remaining authors declare that the research was conducted in the absence of any commercial or financial relationships that could be construed as a potential conflict of interest.
